# Borrowing trouble? The impact of a systematic review service on interlibrary loan borrowing in an academic health sciences library

**DOI:** 10.5195/jmla.2021.1005

**Published:** 2021-01-01

**Authors:** Christy Jarvis, Joan Marcotte Gregory, Alison Mortensen-Hayes, Mary McFarland

**Affiliations:** 1 christy.jarvis@utah.edu, Spencer S. Eccles Health Sciences Library, University of Utah, Salt Lake City, UT; 2 joan.gregory@utah.edu, Spencer S. Eccles Health Sciences Library, University of Utah, Salt Lake City, UT; 3 alison.mortensenhayes@utah.edu, Spencer S. Eccles Health Sciences Library, University of Utah, Salt Lake City, UT; 4 mary.mcfarland@utah.edu, Spencer S. Eccles Health Sciences Library, University of Utah, Salt Lake City, UT

## Abstract

**Background:**

With the mandate to review all available literature in the study's inclusion parameters, systematic review projects are likely to require full-text access to a significant number of articles that are not available in a library's collection, thereby necessitating ordering content via interlibrary loan (ILL). The aim of this study is to understand what effect a systematic review service has on the copyright royalty fees accompanying ILL requests at an academic health sciences library.

**Case Presentation:**

The library created a custom report using ILLiad data to look specifically at 2018 ILL borrowing requests that were known to be part of systematic reviews. This subset of borrowing activity was then analyzed to determine its impact on the library's copyright royalty expenditures for the year. In 2018, copyright eligible borrowing requests that were known to be part of systematic reviews represented only approximately 5% of total filled requests that involved copyright eligible borrowing. However, these systematic review requests directly or indirectly caused approximately 10% of all the Spencer S. Eccles Library copyright royalty expenditures for 2018 requests.

**Conclusion:**

Based on the sample data set, the library's copyright royalty expenditures did increase, but the overall financial impact was modest.

## BACKGROUND

The type and volume of work handled by an interlibrary loan (ILL) department is determined by the needs of institutional patrons and varies in accordance with its users' information demands. Furthermore, changes in scholarly publishing, information discovery, and library service models have the potential to influence the pattern of requests handled by ILL departments. Over the past two decades, libraries have adapted to numerous scenarios that had the potential to dramatically alter ILL workflow, including the transition from print to electronic resources, massive journal cancellations in response to budget constraints, the implementation of discovery systems, and the adoption of pay-per-view (PPV) services [1, 2]. Despite concerns that these changes would have major repercussions for ILL departments, a review of the literature indicated that these fears did not materialize.

In recent years, a growing number of academic health sciences libraries have launched a new service model that focuses on support for systematic review projects at their institutions [3]. Systematic reviews involve comprehensive searches of all available literature. While the number of full-text articles needed for analysis and synthesis varies according to the specific review question, systematic review projects frequently require full-text access to hundreds of articles that are not found in the local collection. When articles are not available via institutional subscription, systematic review teams rely on ILL to obtain articles needed for their review. As a result, library involvement in these projects has been accompanied by uncertainty about the impact that this increased ILL borrowing activity might have on copyright payment fees. Understanding the costs associated with systematic review projects is critical for library administrators who are contemplating the feasibility and sustainability of implementing this service.

While earlier studies provided some insight into emerging trends in library services and their potential impact on ILL transactions, no clear conclusions could be extrapolated and applied to the launching of a systematic review service in order to predict the effect this service might have on ILL borrowing. The authors aimed to add to the literature by sharing the process and results of a study that assessed the impact of implementing a systematic review service on the borrowing activity of the library's ILL department. Of particular interest was the effect on payment of copyright fees triggered by the amount of borrowing required to fulfill the demands of various systematic review projects.

## CASE PRESENTATION

The Spencer S. Eccles Health Sciences Library exists at the center of educational, health care, and research efforts at the University of Utah. Located in the geographic center of the health sciences campus, its staff and resources support the Schools of Medicine and Dentistry; the Colleges of Nursing, Pharmacy, and Health; and the university's hospitals and clinics.

In fall 2016, the Eccles Library partnered with the Population Health Foundation of the Center for Clinical and Translational Science (CCTS) to provide a comprehensive systematic review service. In the three years following the launch of the service, the Eccles Library has collaborated with researchers on dozens of reviews of various types of research, including narrative, scoping, rapid, and systematic reviews and meta-analyses. In 2018, the Eccles Library collaborated with researchers on twenty-two reviews, sixteen of which had reached the stage of full-text article retrieval.

The library's ILL Department, consisting of two full-time staff members and a faculty librarian's partial full-time equivalence (FTE), raised the concern that the library's copyright royalty payments might be impacted as a result of the borrowing activity that would be required to obtain the full-text articles that the systematic review teams needed. To better understand the impact that this newly deployed systematic review service might be having on the library's copyright fees, the authors undertook an analysis of the borrowing activity associated with systematic review projects to determine the extent to which these borrowing requests triggered copyright payments under the guidelines of section 108 of the US Copyright Law, as interpreted by the Commission on New Technological Uses of Copyright Works (CONTU) [4]. These guidelines allow libraries to obtain up to five articles published within the previous five years from a single journal title on an annual basis. Subsequent ILL borrowing requests for articles from that journal require permission from the copyright holder and payment of associated fees.

To quantify the impact of a systematic review service on the library's copyright fees, the authors identified journal titles common to two datasets: the borrowing transactions that were part of a systematic review project and journal titles that had triggered copyright payments during the same time frame. The overlap between the two datasets indicated that the copyright payment fees for particular journals were influenced by the full-text borrowing needs of systematic review projects. The details of each step in this process are described below.

### Copyright eligible borrowing related to systematic review projects

**Figure 1:**
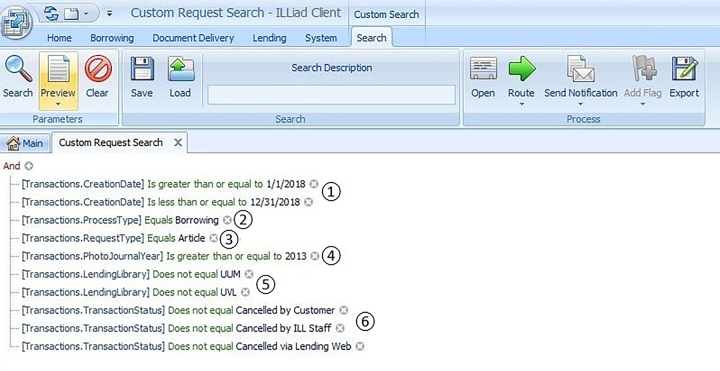
ILLiad search parameters for the custom report

The library's ILL supervisor created a custom report in ILLiad™ [5] to examine borrowing requests that were submitted to the department during the 2018 calendar year for articles published from 2013–2018, because these were the borrowing requests that could have triggered copyright fees in compliance with the CONTU guidelines. While ILLiad allowed the creation of automated reports using Excel formulas for MS Access queries, the ILL team opted to take a manual approach to overcome any limitations imposed by data entry inaccuracies, such as extraneous spaces. To generate the report, the search parameters shown in Figure 1 were used.

The results were exported from ILLiad into Excel. The raw data were copied into a new worksheet for cleanup and formatting, which included the removal of extraneous columns, and the remaining columns were sorted first by request date and then by journal title. The report was saved as “ALL 2018 Copyright Eligible Borrowing Requests.”

The worksheet was then examined to identify the subset of borrowing transactions that were known to be associated with a systematic review project. This was accomplished by looking at the columns indicating ILLiad usernames and corresponding patron names. The library's practice of creating ILLiad usernames unique to each review project allowed staff to isolate ten accounts known to be affiliated with a systematic review project. All data related to these ten usernames were exported into a new Excel spreadsheet that was saved as “S.R. Request Data.” Individual journal titles in this list were color coded to simplify future comparison with the titles composing the entirety of the library's triggered copyright fees in 2018.

### Copyright payment fees

Using the library's copyright.com account, the ILL supervisor retrieved the journal name and International Standard Serial Number (ISSN) of all titles for which the library paid copyright royalty fees related to the Eccles Library's 2018 ILL borrowing activity. This was saved as the “Copyright Journals” Excel spreadsheet. The column of journal titles that had triggered copyright fees was highlighted using the same color-coding scheme used in the “S.R. Request Data” spreadsheet so that the titles could be easily identified after they were exported elsewhere for further comparison work.

### Data comparison

The color-coded set of all journal titles that had triggered copyright fees was then extracted from the “Copyright Journals” spreadsheet and combined with the “S.R. Request Data,” which reflected the journal titles associated with borrowing transactions to support a systematic review project. The color-coding scheme allowed easy identification of titles that appeared on both lists; that is, journal titles that had triggered copyright fees that were also associated with borrowing activity supporting a systematic review project. These borrowing transactions and the associated copyright royalty fees were then evaluated as a percentage of Eccles Library's total copyright fee obligations for 2018 in order to determine the impact of these systematic review projects on the library's copyright fee burden.

The total number of copyright eligible borrowing requests at Eccles Library in 2018 totaled 2,801. Of this number, 155 were known to be associated with a systematic review project, which represented 5.53% of total copyright eligible borrowing for the year. The amount spent on copyright royalty payments for systematic review requests was $167.90, which represented 1.73% of direct copyright costs incurred in 2018.

In addition to these metrics, Eccles Library considered journals that would not have exceeded the copyright threshold had it not been for the systematic review requests. All royalty payments for titles that met this criterion were considered to be indirect copyright costs caused by the library's systematic review service. Taking these titles into consideration, this raised the true cost of systematic review requests to 9.99% of all the library's 2018 copyright expenditures.

Based on the above analysis, Eccles Library concluded that in 2018, copyright eligible borrowing requests that were known to be part of a systematic review represented only approximately 5% of total copyright eligible borrowing requests filled. However, these systematic review borrowing requests directly or indirectly caused approximately 10% of all library copyright royalty expenditures for the year.

## DISCUSSION

Eccles Library was interested in evaluating the impact of a systematic review service on copyright royalty fees. As a result, all borrowing requests that were exempt from such fees were excluded from the analysis. In addition, Eccles Library was only able to associate a borrowing request with a systematic review project where the requestor was known to be working on such a project. The library creates and distributes a single ILLiad user name to all members of a systematic review project at its inception. Any member of the team—including researchers, librarians, and support personnel—can initiate an ILL borrowing request using the shared account information that is associated with the project. As a result of this practice, a list of usernames associated with systematic review projects was available for this study. However, it is possible that some ILL transactions might not have been flagged as being part of a systematic review project if the requester inadvertently used personal ILLiad account credentials rather than the group account. While there is no way to know how often this may have occurred, the librarian team members educate their colleagues about the ILL borrowing procedure and have observed a high rate of compliance with the process. Still, it is not out of the question to assume that systematic review borrowing requests occasionally occur outside the usual protocols. Any such transactions would not be accounted for in this study.

While the implementation of a systematic review service at Eccles Library was accompanied by apprehension about a possible precipitous rise in copyright eligible ILL transactions, the results of this study indicated that such an increase did not, in fact, occur. These findings were in line with those reported by libraries that have confronted scenarios or undergone transitions with a similar potential for increased ILL borrowing activity. Although these circumstances were not strictly equivalent to the ILL needs occasioned by a systematic review service, they were still instructive for the pattern they revealed about anticipated versus actual impact on ILL borrowing.

Perhaps the most analogous scenario can be found in widespread journal cancellations that are necessitated by rising subscription costs and static or shrinking library budgets. In theory, these cancellations have the potential to impact an institution's ILL borrowing activity as patrons seek alternative means for obtaining content that was previously available via library subscriptions. The question of whether this trend of journal cancellations has, in fact, impacted ILL activity has been addressed in the literature. In the 1990s, studies by Crump and Freund [6] and Kilpatrick and Preece [7] concluded that large-scale journal cancellations had minimal impact on the volume of ILL borrowing activity. Similar findings were reported by Calvert, Fleming, and Hill in 2013, where a large-scale cancellation and loss of content led to a mere 2% increase in ILL borrowing [8]. In 2016, Nash and McElfresh noted that journal cancellations at their health sciences library had a negligible impact on their borrowing transactions [9]. Nash and McElfresh's study is particularly relevant to the authors' current investigation because it looked not only at ILL borrowing activity, but also at the impact on copyright fees. Nash and McElfresh concluded that the library did not experience an increase in copyright payments in the wake of their widespread journal cancellations.

Another recent library trend that was expected to have an impact on ILL activity has been the increasing use of various pay-per-view services, such as the Copyright Clearance Center's Get It Now, in which the library covers the cost of individual journal articles as they are requested by patrons. In 2011, Murray State University reported that, one year after the implementation of “transactional access” via a pay-per-view service, the library's ILL department experienced no significant change in its workload and copyright fees [10].

The previous two decades have seen academic libraries transitioning their collections from primarily print-based resources to digitally accessible online resources. This shift had the potential to alter the demand for ILL services. However, a report by Jackson [11] and subsequent studies by Yue and Syring [12] and Rheiner [13] concluded that ILL activity did not experience a consistent downward trend that correlated to the increasing availability of online resources.

Investigation into these library trends—the rise of e-resources, the loss of content due to journal cancellation, and the growth of transactional access—has established that the concerns about rising ILL borrowing transactions and accompanying copyright fees were unfounded. However, it was unknown if this pattern of minimal impact on ILL borrowing would hold true in the context of launching a systematic review service. This knowledge gap concerning the impact of a systematic review service on ILL borrowing and copyright fees led the authors to undertake the present study. An analysis of the data amassed at this academic health sciences library revealed that the impact of a systematic review service on ILL copyright fees was insignificant, following the pattern observed in earlier studies of emerging library trends.

The negligible increase in the Eccles Library's copyright royalty fees after implementing a comprehensive systematic review service is likely attributable to three factors. First, the University of Utah is a research-intensive institution and Eccles Library maintains a robust collection of information resources to support the university's research mission. As a result, much of the content that needed to be evaluated by various systematic review teams was available via institutional subscriptions and backfile purchases, thus decreasing the number of necessary ILL requests. Second, the library's systematic review service is offered only to university faculty, staff, and students. By eliminating external requesters, the library minimizes the likelihood of needing to retrieve full-text content from resources that fall outside the scope of its collection. Third, Eccles Library's systematic review service is a relatively new offering that is primarily utilized by faculty and departments with whom the library has a long-standing relationship built on supporting their information needs. As the Eccles Library's systematic review service is sought out by researchers in emerging disciplines across campus, the library may find that its collection is inadequate to support their systematic review projects without relying heavily on ILL borrowing transactions. A follow-up study in a few years will help Eccles Library answer that question.

The implementation of a systematic review service had minimal impact on the copyright royalty fees accrued by the library's ILL department. These results are most likely to be realized at other institutions that closely mirror Eccles Library in terms of having a robust collection and the ability to take on systematic review work that fits within the scope of existing information resources. As the library seeks to expand the capacity of its existing systematic review service by hiring additional librarians, the current study provides baseline data to help project the financial burden of copyright fees associated with these projects. In addition, repeating this analysis on a yearly basis will allow the authors to observe trends over time and determine if these results remain steady over a longer time frame.

## Data Availability

Data associated with this article cannot be made publicly available because they contain personally identifiable patron information. Access to the data can be requested from the corresponding author at christy.jarvis@utah.edu.
